# A feedback mechanism between PLD and deadenylase PARN for the shortening of eukaryotic poly(A) mRNA tails that is deregulated in cancer cells

**DOI:** 10.1242/bio.021261

**Published:** 2016-12-23

**Authors:** Taylor E. Miller, Julian Gomez-Cambronero

**Affiliations:** 1 Center for Experimental Therapeutics and Reperfusion Injury, Brigham and Women's Hospital and Harvard Medical School, Boston, MA 02115, USA; 2 Department of Biochemistry and Molecular Biology, Wright State University School of Medicine, Dayton, OH 45435, USA

**Keywords:** Mammalian cells, Cell signaling, Gene expression, Breast cancer, MRNA decay, Ribonuclease, Deadenylase

## Abstract

The removal of mRNA transcript poly(A) tails by 3′→5′ exonucleases is the rate-limiting step in mRNA decay in eukaryotes. Known cellular deadenylases are the CCR4-NOT and PAN complexes, and poly(A)-specific ribonuclease (PARN). The physiological roles and regulation for PARN is beginning to be elucidated. Since phospholipase D (PLD2 isoform) gene expression is upregulated in breast cancer cells and PARN is downregulated, we examined whether a signaling connection existed between these two enzymes. Silencing PARN with siRNA led to an increase in PLD2 protein, whereas overexpression of PARN had the opposite effect. Overexpression of PLD2, however, led to an increase in PARN expression. Thus, PARN downregulates PLD2 whereas PLD2 upregulates PARN. Co-expression of both PARN and PLD2 mimicked this pattern in non-cancerous cells (COS-7 fibroblasts) but, surprisingly, not in breast cancer MCF-7 cells, where PARN switches from inhibition to activation of PLD2 gene and protein expression. Between 30 and 300 nM phosphatidic acid (PA), the product of PLD enzymatic reaction, added exogenously to culture cells had a stabilizing role of both PARN and PLD2 mRNA decay. Lastly, by immunofluorescence microscopy, we observed an intracellular co-localization of PA-loaded vesicles (0.1-1 nm) and PARN. In summary, we report for the first time the involvement of a phospholipase (PLD2) and PA in mediating PARN-induced eukaryotic mRNA decay and the crosstalk between the two enzymes that is deregulated in breast cancer cells.

## Abstract (precis)

**Summary:** Cell signaling enzyme phospholipase D2 (PLD2) and its reaction product, phospholipid phosphatidic acid (PA), are involved in mediating PARN-induced eukaryotic mRNA decay.

## INTRODUCTION

The shortening of eukaryotic poly (A) mRNA tails is a key way to repress mRNA translation and to induce transcript turnover. Deadenylation is a principal strategy employed by cells to control mRNA stability and gene expression involved in basic cellular functions, such as development and cell differentiation, as well as in pathological conditions, such as chronic inflammation, cancer and an abnormal DNA damage response (Mazan-Mamczarz et al., 2003; Zhang et al., 2010, 2015; Jalkanen et al., 2014). Cytoplasmic removal of poly(A) tails of mRNA transcripts by 3′→5′ exonucleases (deadenylases) is considered the rate-limiting step for controlled mRNA decay (Wilson and Treisman, 1988a; Shyu et al., 1991; Mitchell and Tollervey, 2000; Funakoshi et al., 2007; Nousch et al., 2013; Wolf and Passmore, 2014). Two well-documented cell deadenylases are the CCR4-NOT transcriptional regulatory complex and the PAN complex. By contrast, a third deadenylase, poly(A)-specific ribonuclease (PARN), which is a member of the RNase D exonuclease family and also degrades poly(A) from the 3′ end (Mian, 1997; Moser et al., 1997; Körner et al., 1998), is lesser known in terms of its own specific functional roles and regulation *in vivo*.

PARN is a divalent metal (Mg^2+^) ion-dependent, poly(A)-specific, oligomeric, processive, and 5′ cap-interacting 3′ exonuclease (Martinez et al., 2000). The main action of PARN in mRNA decay is by exonucleolytic cleavage of poly(A) tails to adenosine monophosphate (AMP) and continued mRNA degradation in the 3′ to 5′ direction (Martinez et al., 2000). PARN targets a group of transcripts that code for cell migration and adhesion factors (Lee et al., 2012), and is implicated in the targeted regulation of protein mRNAs including but not limited to those involved in p53, FAK and ERK/MAPK signaling, Fcγ receptor-mediated phagocytosis and BRCA1 DNA damage response (Devany et al., 2013). Additionally, PARN physically associates with Ago2 in the RNA-inducing silencing complex (RISC) (Zhang et al., 2015), and degrades the oncogenic microRNA, miR-21-CA, allowing PTEN and p53 to function properly as tumor suppressors (Buiting et al., 1999; Copeland and Wormington, 2001; Boele et al., 2014). Different cancers display different levels of PARN expression, and PARN can directly regulate the stability of transcripts for c-myc, c-fos, c-jun and others in the p53 and BRCA pathways. The deadenylase activity of PARN ensures low levels of these mRNAs under normal conditions (Moraes et al., 2006; Cevher et al., 2010; Maragozidis et al., 2012; Devany et al., 2013; Zhang et al., 2015). Recently, PARN deficiency has been shown to be involved in telomere disease and cause dyskeratosis congenital, while telomere shortening and familial pulmonary fibroses are linked to mutations in PARN (Mason and Bessler, 2015; Stuart et al., 2015; Tummala et al., 2015).

Phospholipase D (PLD) is a membrane protein important for the structural integrity of cellular membranes and also for its role in cellular signaling through protein-protein interactions or through the product of enzymatic reaction, phosphatidic acid (PA) (Gomez-Cambronero, 2014). There are several isoforms of PLD, but the most studied have been PLD1 and PLD2. The basic action of PLD is to hydrolyze phosphatidylcholine (PC) into its lipid second messenger, PA, and also choline (Wang et al., 1994). PLD and PA regulate cell adhesion of immune cells to collagen, which involves mechanistic control that coincides with the timing of PLD activity (Zhao et al., 2007; Speranza et al., 2014). PLD2 is implicated in a wide variety of disease states. PLD2 overexpression is connected to breast cancer cell growth, proliferation, and metastasis (Henkels et al., 2013) through interactions between PLD2 and the mammalian target of rapamycin (mTOR) pathway, stabilization of mutant p53 and promotion of factors involved in cancer cell proliferation (Gomez-Cambronero, 2014). The PA produced in PLD hydrolysis reactions is also highly mitogenic and is involved in chemotaxis and cell growth (Gomez-Cambronero, 2014).

Elevated PLD2 expression is known to be a cancer survival signal (Foster, 2004; Rodrik et al., 2005; Hui et al., 2006; Shi et al., 2007; Hatton et al., 2015), but how PLD2 regulates other signaling proteins or how it is regulated at the level of gene expression is not well characterized. Our study showed that the shortening of eukaryotic poly(A) mRNA tails by the deadenylase PARN is mediated by PLD2 and PA. We also present evidence that PLD2/PA regulates PARN expression and that PARN also in turn regulates PLD2 transcript levels in a negative feedback loop that is deregulated in cancer cells.

## RESULTS

### Differential expression of PARN and PLD2 in breast cancer cells compared to non-cancerous cells

**Fig. 1.**
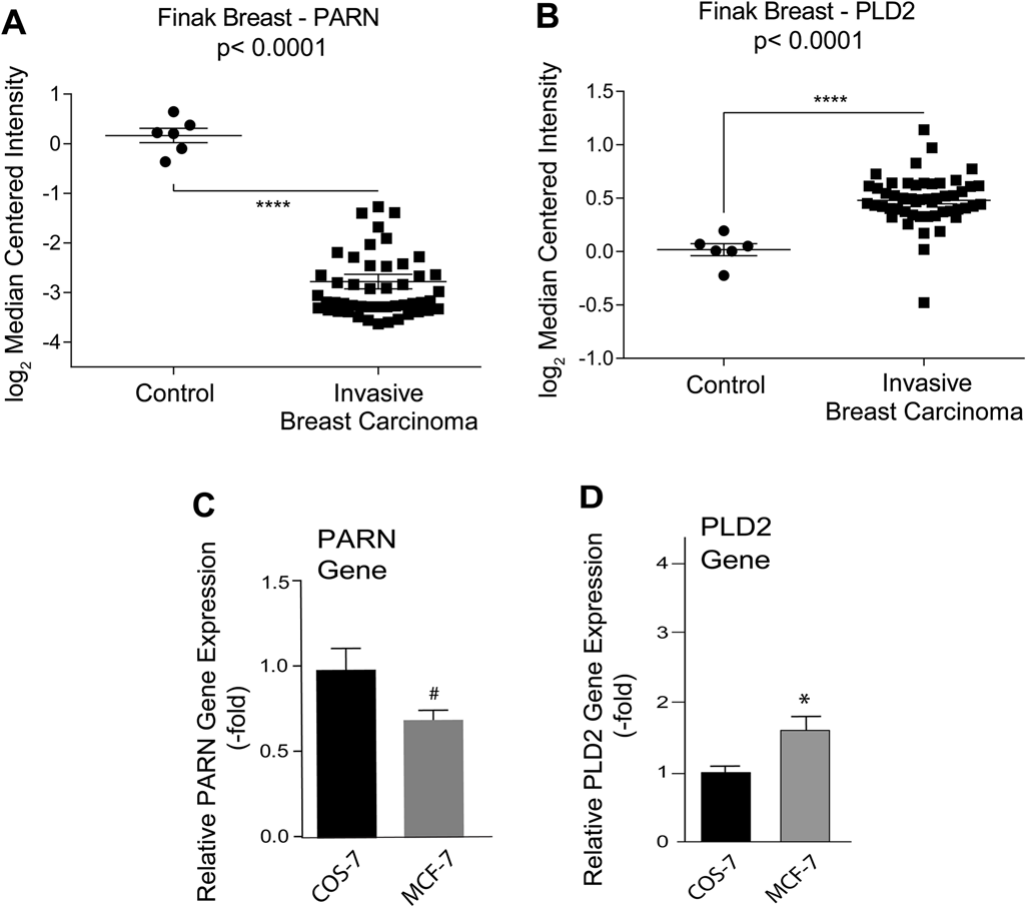
**PARN is differentially expressed in breast cancer versus non-cancerous tissue/cells.** (A,B) Analysis of PARN (A) and PLD2 (B) expression in human breast cancer versus normal adjacent tissue using microarray data from the Finak Breast Dataset (Finak et al., 2008). See Materials and Methods for details. (C,D) Gene expression of PARN (C) or PLD2 (D) in COS-7 and MCF-7 cell lines. Quantitative RT-PCR results in C,D are normalized to the averaged results of the three housekeeping genes used: GAPDH, Actin, and TBP (TATA-binding protein). Data presented as bars are means+s.e.m. Experiments were performed in technical triplicates (for qRT-PCR assays) for *n*=5 independent experiments. The difference between means was assessed by single-factor ANOVA.) *P<0.05, significant increase between samples and controls; #*P*<0.05, significant decrease between samples and controls.

A molecular connection between poly(A) deadenylases and PLD has not been investigated to date. Using the Finak Breast dataset from the ONCOMINE cancer microarray database (Finak et al., 2008), we determined that PARN was downregulated in invasive breast carcinoma compared to adjacent non-cancerous breast stroma (Fig. 1A), while gene expression of PLD2 was significantly upregulated in the same dataset (Fig. 1B). These data suggest that PARN downregulation allowed activation of post-transcriptional events normally kept under its control, which could contribute to cancer development and progression in which PLD2 has already been implicated (Kang et al., 2009; Park and Min do, 2011; Henkels et al., 2013; Kang et al., 2015). We determined the endogenous gene expression of both PARN and PLD2 in COS-7 fibroblasts, used as a non-cancerous ‘normal’ cell line, as well as in the breast cancer cell line MCF-7. As shown in Fig. 1C,D, MCF-7 cells have both significantly lower gene expression of PARN compared to COS-7 and significantly higher gene expression of PLD2, which also reflect the results shown in Fig. 1A,B. The fact that MCF-7 cells had more PLD2 expression than non-cancerous cells was in accordance to previous studies (Fite and Gomez-Cambronero, 2016). However, the fact that MCF-7 cells had less PARN expression than non-cancerous cells was not expected, and as such, we pursued this interesting and potentially novel point further.

### PARN negatively regulates PLD2 expression in non-cancerous cells and PLD2 positively regulates PARN

**Fig. 2.**
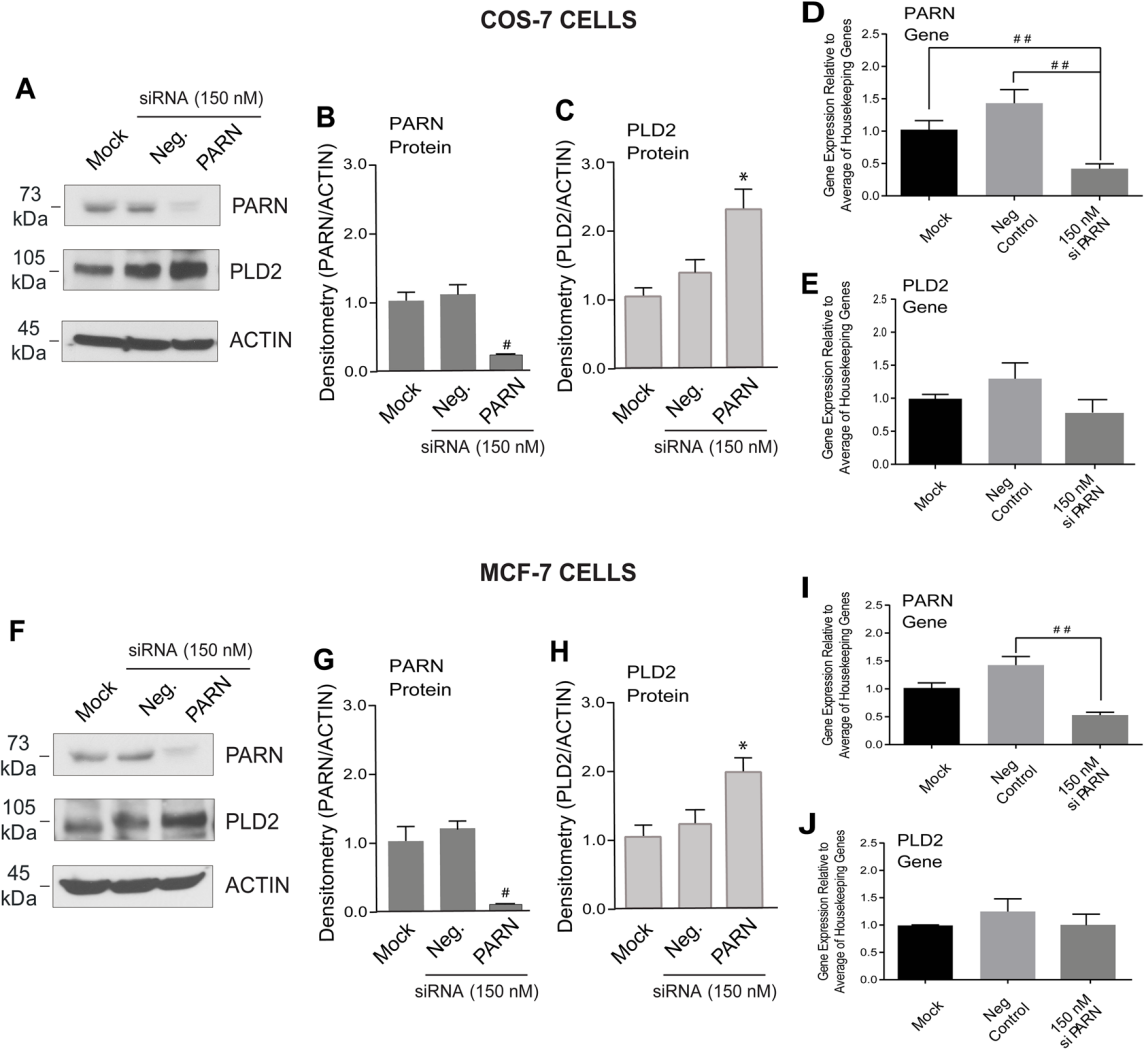
**Silencing of PARN increased PLD2 protein expression.** Cells were treated with transfection reagents only (Mock) or silenced with siRNA-negative control (Neg.) or with siRNA for PARN as indicated. Four days post-transfection, lysates were used for protein and gene expression analyses. (A-C) Protein expression for COS-7 cells and (F-H) for MCF-7 cells. Western blots are presented in A and F and the densitometry of PARN and PLD2 bands are shown in COS-7 cells (B-C) and MCF-7 cells (G-H). Actin was used as the equal protein loading control. (D,E) Gene expression for COS-7 cells and (I,J) for MCF-7 cells measured by RT-qPCR using the three housekeeping genes as indicated in the Fig. 1 legend. Data presented as bars are means+s.e.m. The difference between means was assessed by single-factor ANOVA. *P<0.05, significant increase between samples and controls; ##*P*<0.01, significant decrease between samples and controls.

When PARN was silenced in the non-cancerous COS-7 cell line (Fig. 2A-E), PLD2 protein increased above the mock and negative siRNA-transfected cells (Fig. 2A,C), while PARN protein decreased (Fig. 2A,B). Fig. 2D is the positive control for these experiments, which shows effective silencing of the PARN gene. Interestingly, while PLD2 protein expression increased with PARN silencing, PLD2 gene expression did not change (Fig. 2E), possibly due to faster degradation of a pool of PLD2 mRNA in these non-cancerous cells. In PARN-silenced cancerous MCF-7 cells (Fig. 2F-J), PLD2 protein increased above the mock- and negative control-transfected cells (Fig. 2F,H), while PARN protein (Fig. 2F,G) and gene expression (Fig. 2I) were both silenced. These results indicate that PARN has a strong negative regulatory effect on cellular PLD2 levels.

### Role of the catalytic activities of PLD and PARN

**Fig. 3.**
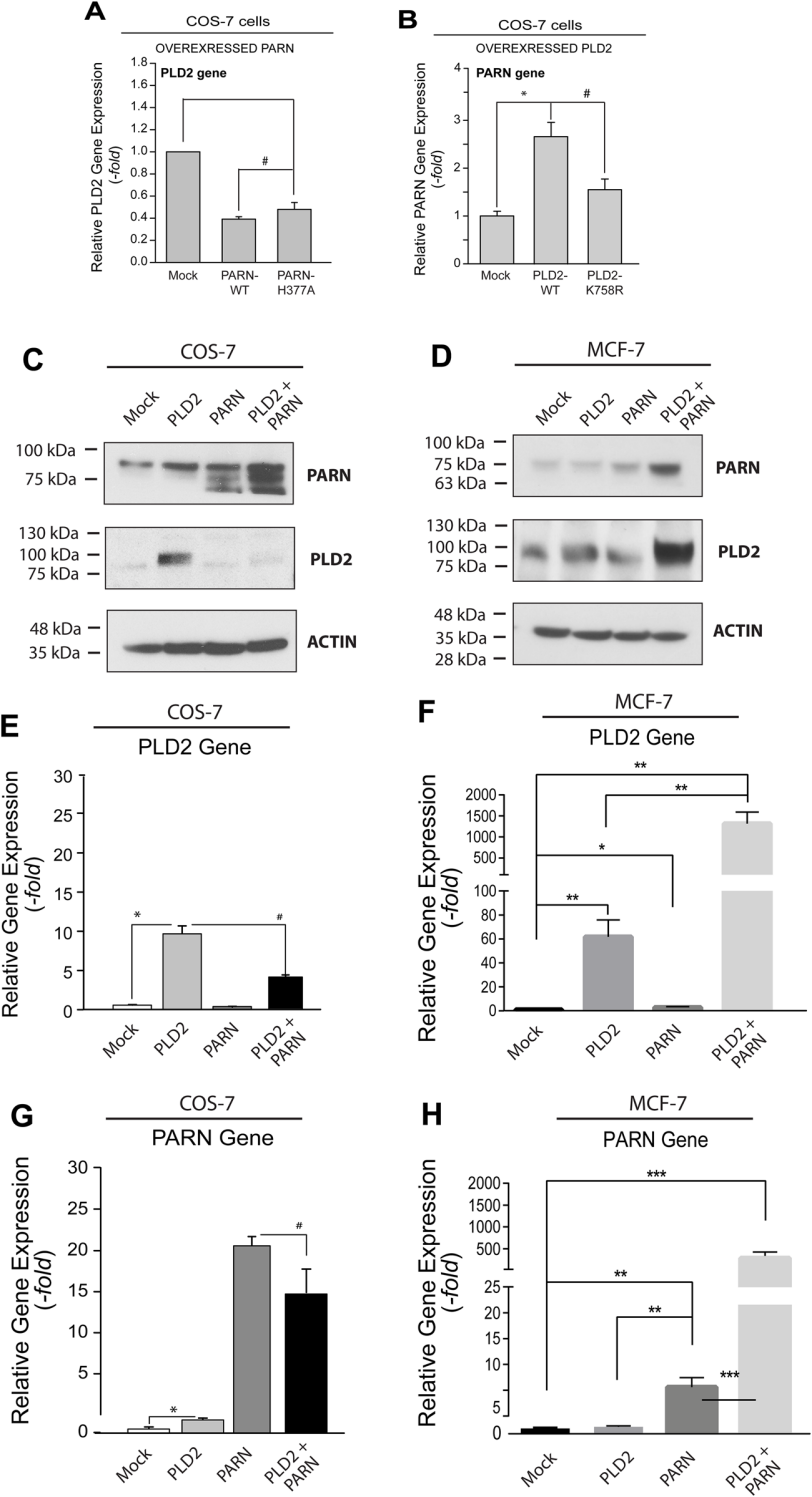
**PLD2 and PARN overexpression affects PARN and PLD2 protein and gene expression.** (A) PARN overexpression of catalytically active or deadenylase-inactive (PARN-H377A) plasmids and their effect on PLD2 gene expression in COS-7 cells. (B) PLD2 overexpression of catalytically active or lipase-inactive (PLD2-K758R) plasmids and their effect of PARN gene expression in COS-7 cells. (C-H) Co-expression of PARN and PLD2 in two different cell lines: COS-7 (C,E,G) and MCF-7 (D,F,H). (C,D) Protein expression by western blot (actin was used as a gel loading control). Cells were left untransfected (mock) or transfected with PLD2 or PARN plasmids individually or in combination (PLD2+PARN). (E,F) PLD2 gene expression and (G,H) PARN gene expression, in either case detected by RT-qPCR. Data presented as bars are means+s.e.m. The difference between means was assessed by single-factor ANOVA. *P<0.05, **P<0.01, ***P<0.005, significant increase between samples and controls; #*P*<0.05, significant decrease between samples and controls.

We next investigated if the catalytic activity of PARN affected PLD2 gene expression. Using a PARN deadenylase-inactive mutant, PARN-H377A, we observed that the ectopic expression of the inactive mutant PARN also decreased expression of PLD2 (Fig. 3A), which was similar to that of wild-type PARN (Fig. 2). This result means that PARN overexpression decreased PLD2 gene expression independent of its deadenylase activity. However, since the PARN-H377A mutant still contains two intact RNA-binding domains, PARN binding to PLD2 mRNA could be destabilized, which could prevent translation of PLD2. Interestingly, when we performed the opposite experiment, one that induced overexpression of PLD2, and then tested the resulting effect on PARN gene expression (Fig. 3B), we observed that the positive effect on PARN gene expression was reversed by overexpression of the lipase-inactive PLD mutant. Thus, the catalytic activity of PARN does not seem to be necessary for PLD2 regulation, but the catalytic activity of PLD is necessary for PARN regulation.

### Differential effects of PARN+PLD2 co-expression in non-cancerous cells versus breast cancer cells

Since a robust signaling interaction between PARN and PLD2 seems to exist in non-cancerous and cancerous cells, we next transfected both types of cells with a combination of expression plasmids coding for both the PARN and PLD2 proteins. Co-expression of PARN+PLD2 in COS-7 cells led to an overabundance of PARN protein expression with a concomitant abrogation in PLD2 protein expression (Fig. 3C). Surprisingly, in the MCF-7 breast cancer cells, both PARN and PLD2 protein co-overexpression augmented the expression of each of these proteins in parallel (Fig. 3D). The results of RT-qPCR assays to measure gene expression are shown for PLD2 in Fig. 3E,F and for PARN in Fig. 3G,H, which also reinforce the protein expression results shown in Fig. 3C,D, in that the expression of PLD2 was at its highest in MCF-7 cancer cells when co-expressed with PARN. Since PLD2 levels are naturally high in cancer cells (Hui et al., 2006; Zheng et al., 2006; Shi et al., 2007), our results indicate that either PARN is deregulated in cancer cells, not allowing inhibition of PLD2 expression, or that PLD2 activity contributes to increased mRNA stabilization, or both. Such synergism would be advantageous for a cancer cell, as PLD is implicated in cell invasion and cancer metastasis (Knoepp et al., 2008; Chen et al., 2012; Henkels et al., 2013)

### Phosphatidic acid increased both PARN and PLD2 protein and gene expression

**Fig. 4.**
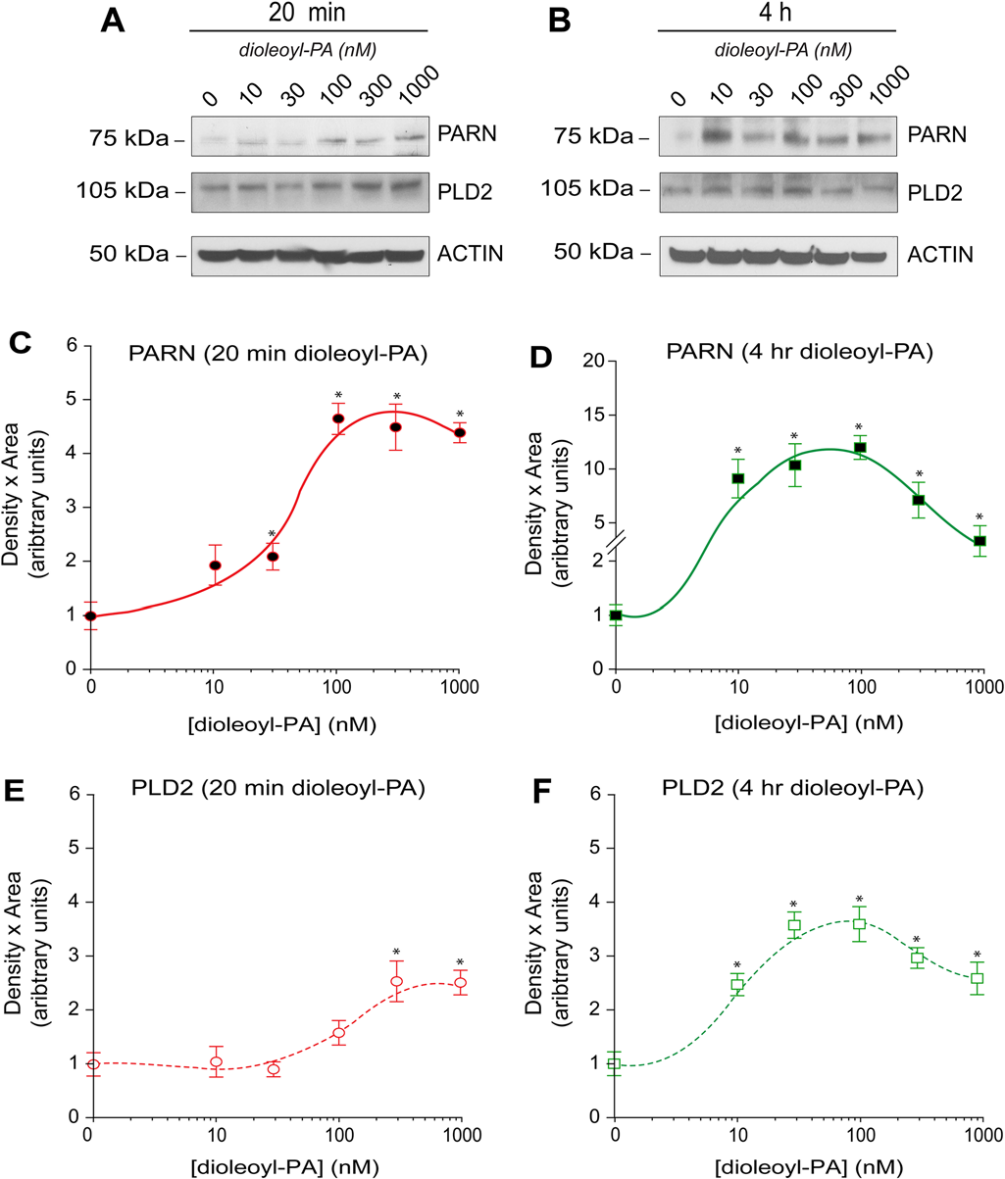
**Exogenous dioleoyl-PA increases PARN protein expression.** (A,B) Western blots showing endogenous levels of PARN, PLD2 and actin protein in COS-7 cells in response to 20 min (A) or 4 h (B) incubation with increasing concentrations of dioleoyl-PA as indicated. (C-F) Densitometry analyses of data shown in A,B. (C,D) Results of densitometry of PARN bands from western blots similar to the ones shown in A,B for 20 min (C) and 4 h (D). (E,F) Results of densitometry of PLD2 bands from western blots similar to the ones shown in A,B for 20 min (E) and 4 h (F). Data are presented as means+s.e.m. *P<0.05 by single-factor ANOVA.

As PLD had a positive effect on PARN expression (Fig. 3C-H) that was negated by a catalytically inactive form of PLD (Fig. 3B), we investigated what effect PA (the product of PLD reaction) might have on PARN. PA is known to be a strong mitogen and regulator of gene transcription (Yoon et al., 2015). COS-7 cells were incubated with increasing concentrations of 1,2-dioleoyl-*sn*-3-phosphate (dioleolyl-PA) for either 20 min or 4 h. As shown in Fig. 4A,B, both PLD2 and PARN protein expression increased concomitantly with PA concentration. Quantification of protein bands are shown in Fig. 4C-F. Both PLD2 and PARN protein expression increased during this 4 h incubation up to a peak at ∼100 nM dioleolyl-PA followed by a decrease at higher concentrations of dioleolyl-PA (Fig. 4D,F). This effect was shifted to higher concentrations of dioleolyl-PA during a shorter incubation time (20 min) (Fig. 4C,E). These results indicate that there is a concentration- and time-dependent threshold to which the cells produce more protein in response to PA that could be reversed.

### Phosphatidic acid stabilized mRNA decay and counteracted an enhanced mRNA degradation rate caused by PARN overexpression

**Fig. 5.**
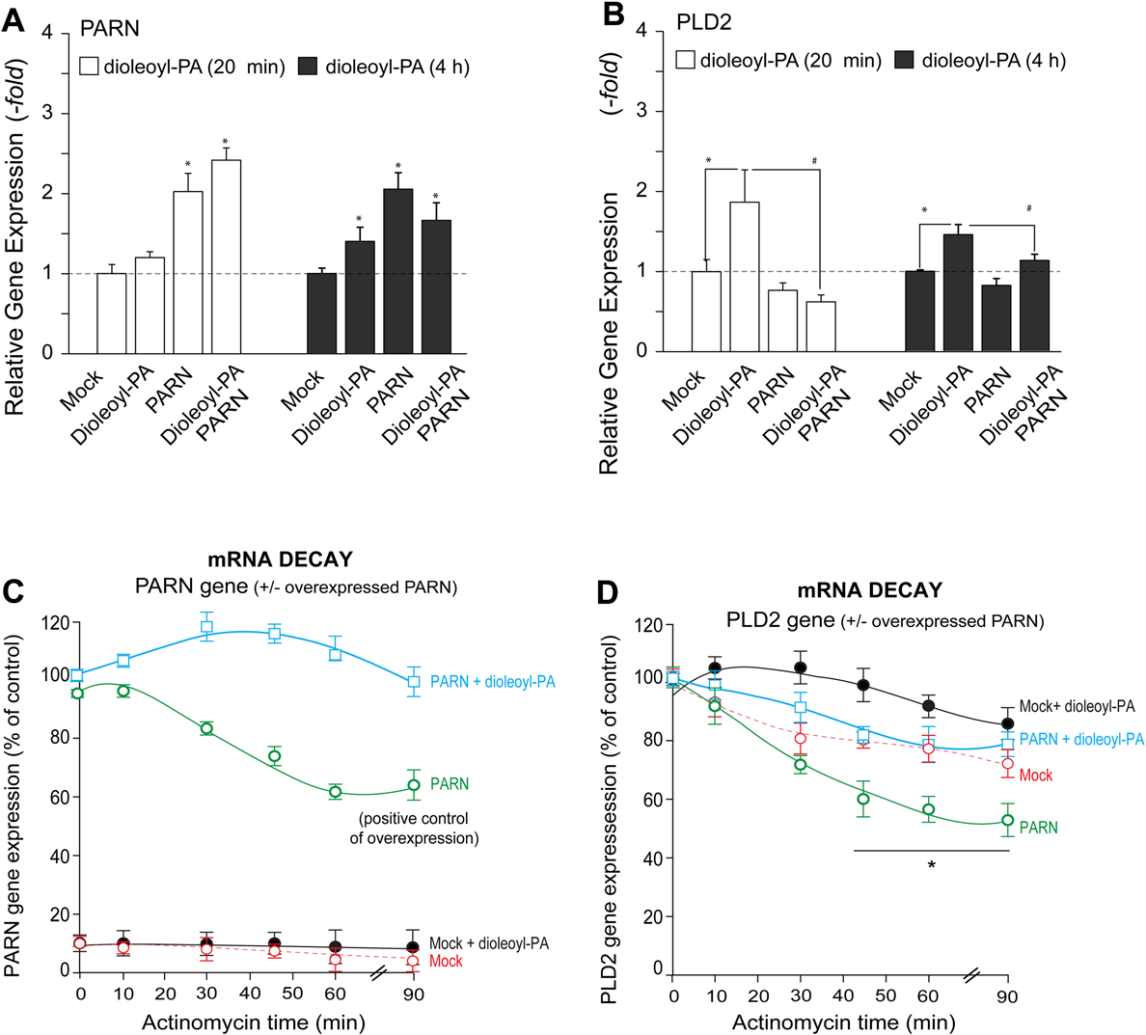
**Effect of dioleolyl-PA treatment on ectopic expression of PARN and mRNA decay.** (A,B) COS-7 cells were transfected with PARN or left untrasnfected (Mock) for 48 h. On the day of the experiment aliquot samples were treated with 300 nM dioleoyl-PA in culture for 20 min (white bars) or for 4 h (black bars). Cells were processed for the measurement of gene expression by RT-qPCR for either PARN (A) or PLD2 (B). Data in A,B presented as bars are means+s.e.m. The difference between means was assessed by single-factor ANOVA. *P<0.05, significant increase between samples and controls; #*P*<0.05, significant decrease between samples and controls. (C,D) mRNA decay study. COS-7 cells were overexpressed with PARN or left untransfected (Mock) for 48 h and incubated with 50 nM actinomycin D on the day of the experiment, for the indicated lengths of time, in the absence or presence of 300 nM dioleoyl-PA. RNA was extracted and then used for qRT-PCR analyses. Relative gene expression was used to determine the levels of mRNA decay for PARN (C) and PLD2 (D) and are expressed in the graphs in terms of mean percentage of control (normalized as 100)±s.e.m. *P<0.05 by single-factor ANOVA.

PARN is one of the main deadenylases that regulates the amount of mRNA at a given time in the cell, thus having a profound effect on mRNA decay (Utter et al., 2011). We have determined that dioleolyl-PA treatment in the absence and presence of overexpressed PARN has positive effects on PARN mRNA levels (Fig. 5A), while PARN negated this positive effect of dioleolyl-PA (Fig. 5B). We next studied mRNA decay. Fig. 5C,D show that actinomycin treatment of cells arrested RNA transcription as a function of time. As expected, PARN mRNA decay was observed in Fig. 5C, while PLD2 mRNA decay was observed in Fig. 5D (dashed red lines for both genes). Not surprisingly, overexpression of PARN accelerated PLD2 mRNA decay (Fig. 5D, green lines). However, this was reversed by the addition of dioleyl-PA to the culture media (Fig. 5D, blue and black lines). This mRNA decay protection afforded by dioleoy-PA was also observed in samples that overexpressed PARN (Fig. 5C, blue lines). These data support a PLD-PA function that stabilized mRNA and protected against degradation as a way of counteracting the enhanced mRNA degradation rate caused by PARN overexpression, which could play an even major role in cancerous cells.

### Exogenous phosphatidic acid added to cell cultures slightly enhances PARN deadenylase activity, as measured *in vitro*

**Fig. 6.**
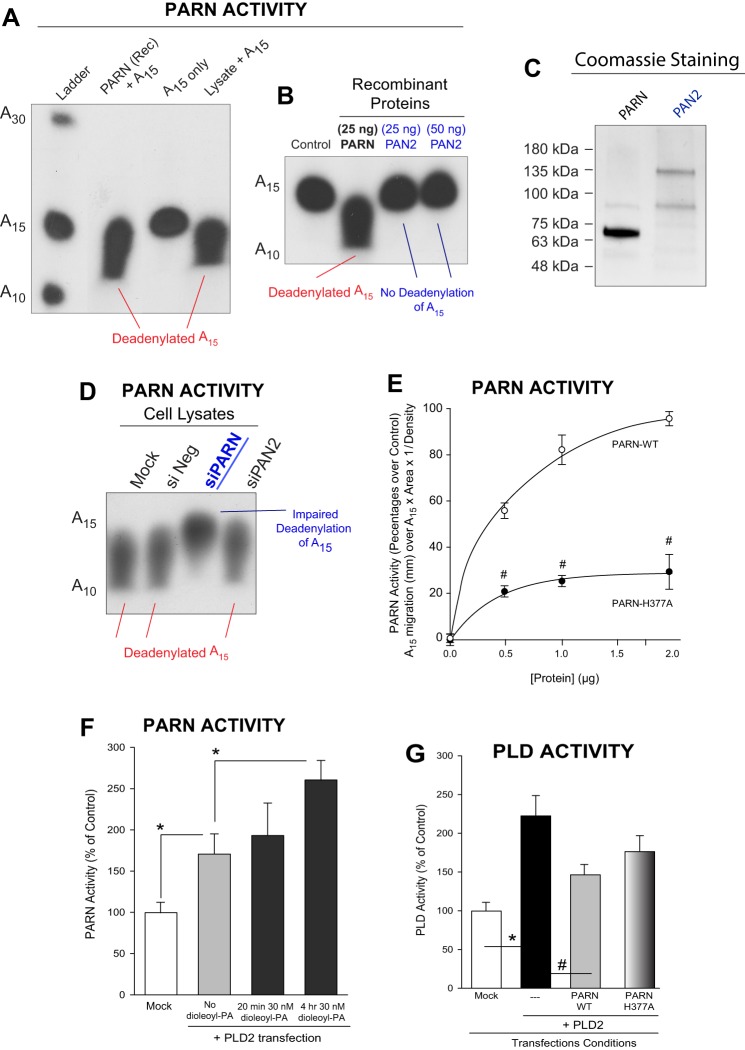
**Effect of PLD or dioleoyl-PA on PARN deadenylation activity.** (A,B) Validation study for *in vitro* PARN deadenylase activity. (A) Radiolabeled A_15_ RNA substrate was deadenylated by recombinant PARN with respect to the A_15_-only control. Deadenylation is evidenced by a greater mobility of radiolabeled spots and the appearance of smears versus the negative control of A_15_ alone. (B) Recombinant PARN, but not recombinant PAN2 is able to deadenylate A_15_. (C) Coomassie-stained gel indicating the high purity of the recombinant, purified proteins used. (D) PARN deadenylase activity as measured in lysates from COS-7 cells that were silenced with 150 ng of either control RNA (SiNeg), siPARN RNA or siPAN2 RNA. (E) PARN activity in overexpressing cells was concentration dependent from cell lysates in comparison with the deadelynase-inactive mutant PARN-H377A. (F) PARN activity of lysates prepared from COS-7 cells overexpressing PLD2 incubated with or without dioleolyl-PA. (G) PLD activity of COS-7 cells overexpressing PLD2 alone (control) or co-overexpressed with PARN-WT or the PARN mutant. Data are presented as means+s.e.m. The difference between means was assessed by single-factor ANOVA. *P<0.05, significant increase between samples and controls; #*P*<0.05, significant decrease between samples and controls.

We next investigated if PA affects the deadenylase activity of PARN. To accomplish this goal, we set up a robust and reliable *in vitro* enzymatic PARN deadenylase assay, validated in Fig. 6A-C. In Fig. 6A, [^32^P]-γATP-radiolabeled A_15_ RNA substrate was deadenylated by recombinant PARN with respect to the A_15_-only control. Deadenylation was evidenced by an increased mobility of radiolabeled and degraded products (the smeared product) versus the input A_15_ negative control alone. Fig. 6B shows that recombinant, purified PARN protein but not recombinant, purified PAN2 protein (another closely related deadenylase, as stated in the Introduction) deadenylated the A_15_ substrate. Fig. 6C shows a Coomassie-stained gel that indicates the high purity of the recombinant, purified proteins used. PARN deadenylase activity was effectively silenced in cells with siPARN RNA (Fig. 6D) but not with siPAN2 RNA. This indicates that in our assay conditions, PAN2 did not contribute to the deadenylase activity found in cell lysates.

A further control for these activity experiments is shown in Fig. 6E, whereby PARN activity in lysates from cells overexpressing wild-type PARN increased in a concentration-dependent manner when compared to overexpression of the deadelynase-inactive mutant PARN-H377A. Dioleoy-PA at 30 nM slightly affected deadenylase activity of PARN in PLD2-overexpressing cells (Fig. 6F), although the increase in PARN mass may end up cancelling that effect. Further, PLD activity (Fig. 6G) was enhanced by dioleoyl-PA but the combination of dioleoyl-PA+ PARN overexpression proved once again to negatively impact PLD activity (as in Fig. 5B for gene expression). Taken together, these data indicate that PARN overexpression negatively affects PLD lipase activity, and PLD2 positively affects PARN expression and activity.

### PARN localizes to PA-containing vesicles

**Fig. 7.**
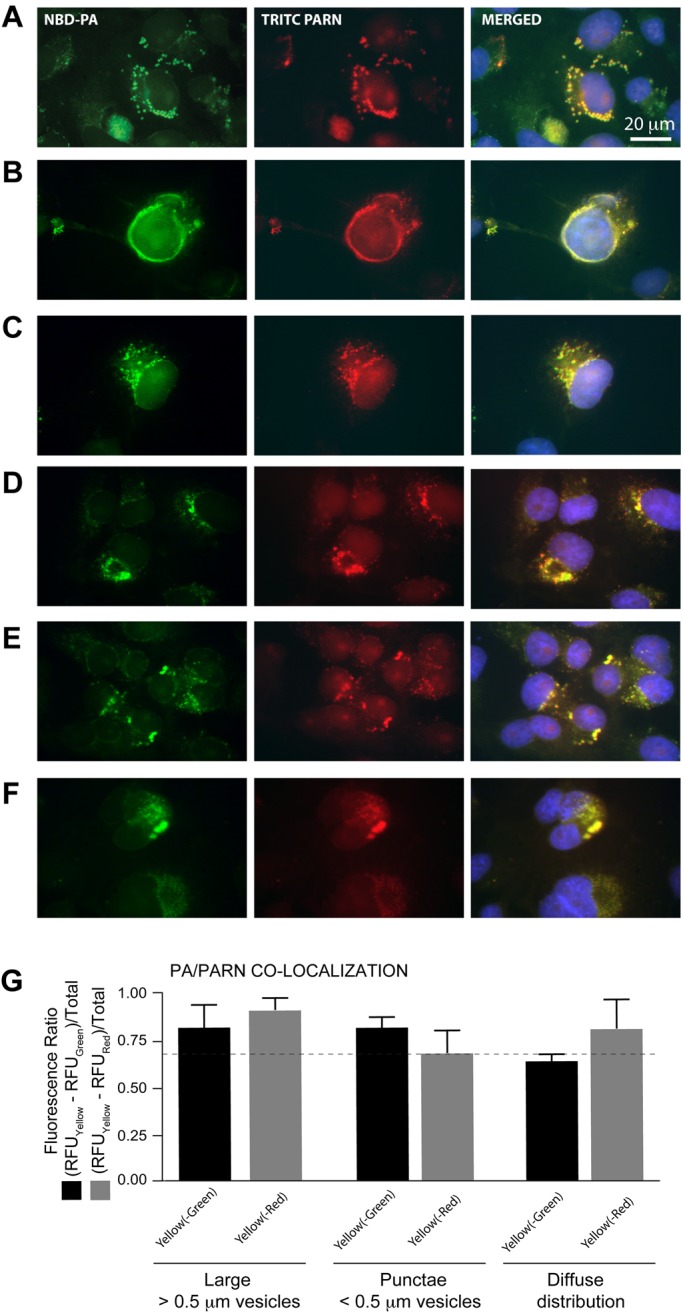
**Co-localization of PARN with NBD-PA.** COS-7 s were incubated for 30 min in 30 nM fluorescent PA (NBD-PA) and were then used for immunofluorescence microscopy using TRITC-conjugated α-PARN IgG antibodies. (A-F) Sextuplicate fields. Localization of the NBD-PA is in green (excitation=490 nm; emission=525 nm, using a FITC filter) and localization of PARN is in red (excitation=557 nm; emission=576 nm, using a TRITC filter). Nuclei were stained blue with DAPI and the images merged. (G) PA/PARN co-localization, represented by the presence of yellow (550 nm) immunofluorescence, as indicated in Methods, in large vesicles (>0.5 mm); punctae (<0.5 mm) or diffuse distribution. This classification of vesicles is in accordance with a previous publication from our lab (Henkels et al., 2016). The dashed line marks the threshold for ratios of least 70% of maximum values. Each bar is the average of the six images shown plus other three fields not shown, for a total of *n*=18. Data are presented as means+s.e.m.

The level of PLD2 and PARN interregulation we have documented suggests that these two proteins interact with one another and could potentially be in close spatial proximity. To test this hypothesis, we utilized a fluorescently-tagged form of PA, green fluorescent 1-oleoyl-2-{6-[(7-nitro-2-1,3-benzoxadiazol-4-yl)amino]hexanoyl}-*sn*-glycero-3-phosphate (NBD-PA) that could be easily incubated with cultured cells. Subsequent immunofluorescence microscopy of such cell samples could reveal the potential co-localization of fluorescently-labeled PARN with the NBD-tagged PA. In our experimental design, PARN was stained red in these samples using anti-PARN-TRITC IgG antibody labeling, NBD-PA was green and DAPI-stained nuclei were blue. As shown in Fig. 7A-F, the green NBD-PA localized diffusely throughout the cytoplasm of the COS-7 cells but also localized very strongly in vesicles near the nucleus. PARN was observed inside the nucleus, particularly in the nucleoli, and also in the NBD-PA-containing vesicles (as seen by the strong yellow signal in the merged images).

Quantification of the co-localized PARN+NBD-PA signals (Fig. 6G) indicates that PARN co-localized with PA to large vesicles (>0.5 mm) to a very large extent (close to 90%) and to punctate vesicles (<0.5 mm) (close to 75%). We have observed a similar localization pattern for PA in cells in a previous study that focused on the role of PA as an intracellular transport conveyor for the epidermal growth factor receptor (EGFR) (Henkels et al., 2016). We believe that trafficking of intracellular PA assists in mediating the effects of PARN on PLD2, especially in regards to PARN deadenylation activity and PLD2 mRNA decay.

### A model of regulation between PLD2/PA and PARN

**Fig. 8.**
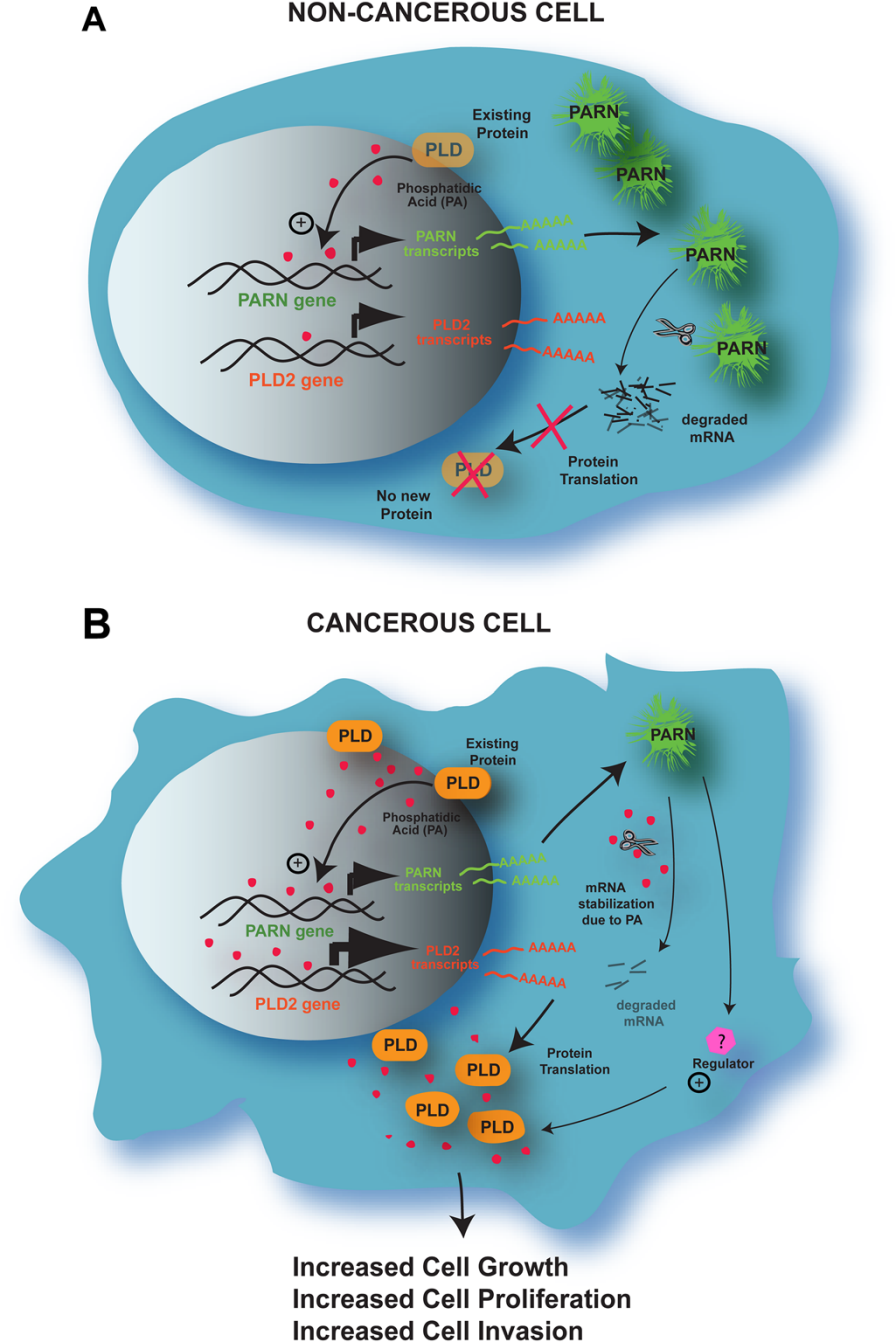
**Proposed model for the interactions of PARN with PLD2 in non-cancerous versus cancerous cells.** (A) In non-cancerous cells, where PARN expression surpasses PLD2 expression, a positive and a negative feedback mechanism exists, whereby PLD2 and PA production initially upregulates PARN gene and protein expression. This PARN protein then decreases PLD2 expression by degrading PLD2 mRNA bringing PLD2 levels back to a normal level. (B) Working model for the deregulation between PARN and PLD2 in cancerous cells, where PLD2 expression surpasses PARN expression. As in non-cancerous cells, PLD upregulates PARN. However, PARN can not downregulate PLD. Either degradation of mRNA is compromised by an stabilizing effect of PA or a positive regulator exists between PARN and PLD protein translation (yet to be established), leads to higher PLD2 protein expression, which mediates the indicated functionality of these cells.

A model representing the proposed regulation we have characterized between PLD2/PA and PARN is presented in Fig. 8. The results of our novel study indicate that eukaryotic mRNA decay was regulated by PARN and the activation and synthesis of PARN was enhanced by phosphatidic acid (PA), the hydrolysis product of phospholipase D2 (PLD2). When PLD2/PA levels were increased in non-cancerous cells, like COS-7, this caused an increase in PARN gene and protein expression, as well as an increase in its deadenylase activity. This overall elevation in PARN levels in the cell then decreased PLD2 expression to basal levels. A negative feedback mechanism exists in cells, whereby PLD2 overexpression and PA production positively affected PARN gene and protein expression, while PARN overexpression negatively affected PLD2 gene and protein expression. The large negative feedback of PARN on PLD expression was attenuated in cancer cells, perhaps due to the effect of an as-yet-unidentified regulator (shown in Fig. 8), which supports the significant role of PLD in this disease, and relates to the relative higher level of cell invasiveness and metastatic potential found in breast cancers.

## DISCUSSION

This work identifies that the phospholipid signaling enzyme PLD2 and its catalytic product, PA, are a novel route for modulating PARN-mediated mRNA decay in higher eukaryotes. We report that a possible inter-regulatory relationship exists, whereby PLD2 affected PARN gene and protein expression, as well as activity and vice versa. This mechanism appears to be modulated differently in non-cancer versus cancer cells. The degradation of many mRNAs is initiated by poly(A) tail shortening and can be accomplished by (1) the PAN complex system, (2) the CCR4/NOT complex (Basquin et al., 2012) and (3) by the herein-reported PA/PLD2-regulated PARN. The results shown here support an important role for PA and PLD2 in PARN mRNA decay and deadenylation of mRNA transcripts, independently of the PAN and CCR4/NOT complexes.

The relationship between phospholipases and RNases is interwoven in terms of cellular location and function. Previous studies have linked phospholipase C (PLC) to RNase activity, which can be used as a basis for the possibility of other phospholipases being involved in RNase activities, such as PLD2/PA-regulated PARN. It has been previously determined that PLD and ribonucleases co-localize in the vacuoles of plants (Matile, 1968). It is already known that PARN exists both in the cytoplasm and nucleus, whereas PLD2 has been shown to be in the cytoplasm, while PA, the important secondary messenger product of PLD2, can be found throughout the cell to exert a variety of functions in cell signaling. (Zini et al., 1989; Hondal et al., 1997; Kravchuk et al., 2001). Taken together, the research on PLC and understanding the subcellular localization of PLD2, PA and PARN, support the notion that these molecules have ample opportunity and probability of direct and/or indirect interaction.

What purpose does the dual feedback in cancer cells serve? By using normal and breast cancer cell lines that were mock-treated or that overexpressed PARN and/or PLD2, we defined the relationship between PARN and PLD2. First and foremost, cancer cell lines have increased levels of PLD2 and therefore higher levels of PA production (Foster, 2009; Foster et al., 2014). The product of PLD hydrolysis of membrane PC is almost exclusively monounsaturated PA (e.g. dioleoyl-PA), and as such we have used exogenously added dioleolyl-PA in our experiments. Our exogenously added PA stabilized both PARN and PLD2 mRNA degradation through the partial or total inhibition of mRNA decay. We believe that a negative feedback mechanism exists in cells, whereby PLD2 overexpression positively affected PARN gene and protein expression, while PARN overexpression negatively affected PLD gene and protein expression. This negative feedback mechanism between PARN and PLD2 was diminished in our breast cancer cell line, MCF-7, possibly due to an as-yet-unidentified regulator or pathway that leads to the attenuation of the large negative feedback of PARN on PLD2 expression, further supporting the significant role of PLD in this disease, as relates to the relative level of cell invasiveness and metastatic potential. Cancer cells having low levels of RNase could potentially maintain a more active pool of RNA and facilitate overexpression of proteins like PLD2 that are highly relevant to cell migration and metastasis.

Regarding the mechanism of how PA inhibited PARN-induced mRNA decay, we considered the previous work on polycationic aminoglycoside antibiotics (Hermann and Westhof, 1998; Woegerbauer et al., 2000). These antibiotics inhibit PARN activity, although the possibility of steric hindrance must also be considered. Our data on the inhibitory effect of PA on PARN takes into account a potential direct effect on the enzyme itself, not on RNA, as PA is negatively charged. It is possible that the mechanism of the inhibition of PARN-induced mRNA decay by anionic PA is that PA displaces catalytically essential divalent metal ions in the active site of PARN that diminishes the processivity of the deadenylation reaction. PARN was initially identified as a Mg^2+^-dependent exonuclease (Astrom et al., 1991; Körner and Wahle, 1997; Copeland and Wormington, 2001), and our findings are in agreement with reports on DNaseI (Woegerbauer et al., 2000) and PLC (Burch et al., 1986).

In conclusion, we report here a regulatory relationship between PARN and PLD2/PA, which constitutes a new and possibly essential component in post-transcriptional regulation in higher eukaryotes, as well as in disease states such as cancer. Further investigation is needed into determining how and why the interaction between these two proteins becomes dysregulated in breast cancer to develop methods for re-establishing normal interaction and remedying or preventing the cancerous pathology.

## MATERIALS AND METHODS

### Materials

Rabbit PLD2 (N-term) (Abgent AP14669a, San Diego, CA), rabbit Pierce PARN (Thermo Scientific PA5-30252, Pittsburgh, PA), rabbit beta-actin (Cell Signaling 4970S), and HRP-linked rabbit IgG (Cell Signaling 7074S) antibodies were used for western blots. For immunofluorescent microscopy, goat PARN (N-12) (sc-47618) and donkey anti-goat IgG-R (sc-2094) were purchased from Santa Cruz Biotechnology (Dallas, TX). Antibodies were validated by the manufacturer. 1,2-dioleoyl-*sn*-3-phosphate (dioleolyl-PA; 840875) and green fluorescent 1-oleoyl-2-(6-[(7-nitro-2-1,3-benzoxadiazol-4-yl)amino]hexanoyl)-*sn*-glycero-3-phosphate (NBD-PA; 810175) were purchased from Avanti Polar Lipids (Alabaster, AL). Transit 2020 transfection reagent was from Mirus Bio (Madison, WI). ECL western blotting reagents were from GE Healthcare (Piscataway, NJ). qPCR reagents and enzymes were from Fisher Scientific/Life Technologies (Pittsburgh, PA). Plasmid DNAs used herein were as follows: pcDNA3.1-myc-PLD2-WT and pcDNA3.10-myc-PLD2-K758R were previously designed in our lab, pCMV6-myc-DDK-PARN-WT was from Origene (RC207220, Rockville, MD) and this plasmid was the basis for site-directed mutagenesis to generate PARN-H377A. Ambion single siRNA were purchased from ThermoFisher Scientific and were as follows: 5 nM PARN siRNA (AM16708 ID#: 11661), 5 nM PAN2 siRNA (|AM16708A ID#: 105177), and 50 µM Negative Control siRNA (AM4611). Pooled siRNA were purchased from Santa Cruz Biotechnology and were as follows: 10 µM PARN siRNA (sc-61297) and 10 µM control siRNA-A (sc-37007). Recombinant purified PARN (TP307220) and PAN2 (TP300573) were purchased from Origene.

### Cells and cell culture

COS-7 and MCF-7 cells were obtained from ATCC (Manassas, VA, USA). The repository has indicated that the cell lines were recently authenticated and tested negative for contamination. DMEM was from HyClone and supplemented with 10% (v/v) FBS. Cells were maintained at 37°C in an incubator with a humidified atmosphere of 5% CO_2_. COS7 and MCF-7 cells were cultured in Dulbecco's modified Eagle's medium (DMEM) supplemented with 10% (v/v) fetal bovine serum (FBS).

### ONCOMINE cancer microarray database

Using the ONCOMINE cancer microarray database (www.oncomine.org) as an integrated data-mining tool, we compared the gene expression profiles of PARN and PLD2 in *n*=53 human tumor stoma samples contrasted against *n*=6 normal breast stroma samples. The Finak Breast dataset (Finak et al., 2008) analyzed subtypes of tumor stroma corresponding to good- and poor-outcome breast cancers from invasive breast cancer patients. The data link website for the Finak Breast Dataset Summary can be found at https://www.ncbi.nlm.nih.gov/geo/query/acc.cgi?acc=GSE9014, which is maintained by the NCBI.

### Dioleoyl-PA and NBD-PA incubation

‘Super-Stock’ 1 mM dioleoyl-PA was prepared with 1 mg of 1,2-dioleoyl-*sn*-3-phosphate, or NBD-PA, in 1.4 ml of Super-Stock Buffer consisting of 50 mg of fatty acid-free BSA per 10 ml of 1× PBS, pH 7.2. Dioleoyl-PA is a cell permeable form of phosphatidic acid (PA) (Lehman et al., 2007). This Super-Stock dioleoyl-PA was then sonicated on ice 2×4 s each with a 4 s pause in between sonications. ‘Intermediate 100 µM liposomes’ were then made using 25 µl of the Super-Stock dioleoyl-PA and 225 µl of cell starvation media (DMEM+0.1% bovine serum albumin), which were then used to prepare the final concentration of 30 nM (unless otherwise indicated by the figure legends) dioleoyl-PA used to incubate cells for the indicated times in various figures.

### Actinomycin D experiments

COS-7 cells were plated into 6-well plates and transfected with 1 µg PARN-WT or left untreated. The day of the experiment, all cells were starved for 2 h in cell starvation media. To test the effect of dioleoyl-PA, 300 nM dioleoyl-PA in cell starvation media was added to relevant wells for 20 min or 4 h. After the specified incubation time, the dioleoyl-PA solution was removed and 50 nM actinomycin D in cell starvation media was added. After each time point (0, 10, 30, 45, 60, and 90 min), the actinomycin D solution was aspirated from the cells, wells were gently washed twice with 1× PBS, trypsinized, and cells pelleted for use in qRT-PCR to measure PLD2 and PARN gene expression. For each respective gene (PARN or PLD2), PARN overexpressed plus dioleoyl-PA at time 0 was set to 100% and all other samples were calculated and graphed relative to this point.

### Cell transfection

Cells were plated into 6-well plates in complete media and allowed to grow 12-24 h before transfection. All overexpression transfections were accomplished using 2 µg of PLD2 plasmid and/or 1 µg of PARN plasmid, 300 µl Opti-Mem serum-free media, and 2 µl of Transit 2020 transfection reagent per 1 µg DNA. For silencing, both the single and pooled PARN and PAN2 siRNAs purchased were diluted with the manufacturer's included nuclease free water to a stock concentration of 50 µM. These siRNA solutions were then further diluted to 150 nM for use in all silencing experiments. Equal concentration of negative control siRNA was used in the negative control siRNA transfections. The siRNA reactions consisted of siRNA, 300 µl Opti-Mem serum-free media, and 5 µl Dharmafect reagent. All reactions were incubated at room temperature for 20 min before being added drop-wise to the corresponding cells growing in complete media. Cells were then incubated at 37°C in an incubator with a humidified atmosphere of 5% CO_2_ for 24 h. Media was aspirated and fresh complete media added and cells allowed another 24 h of growth in the incubator before harvesting.

### SDS-PAGE/western blotting

COS-7 and MCF-7 cells were transfected as described previously in these Materials and Methods with expression plasmids that are defined in the figure legends. Cell media was aspirated from the plates and cells washed twice gently with 1× PBS. Cells were then lifted from the plates by incubation with the addition of 1 ml trypsin for no more than 5 min, collected into 1.5 ml conical snap-cap tubes, and sedimented at 14000 ***g*** 4°C for 1 min. The supernatant was aspirated and cell pellets were suspended in special lysis buffer (SLB; 5 mM HEPES, 1 µM leupeptin, 768 nM aprotinin, 100 µM sodium orthovanadate and 0.4% Triton X-100). After sonication of the lysates, samples were resolved using SDS-PAGE and transferred to a PVDF membrane, followed by immunoblot analysis with anti-PARN (1:2000 dilution), anti-PLD2 (1:500 dilution), and anti-actin (dilution 1:3000) antibodies and visualized using ECL reagents. Actin was used as equal protein loading control.

### Coomassie staining

Approximately, 100 ng of purified, recombinant PARN and PAN2 protein were run on gels using standard SDS-PAGE protocol. The gel was then rinsed three times for 5 min each in purified distilled water then incubated overnight in 20 ml of GelCode Blue Safe Protein Stain (Thermo Scientific, 1860983) with gentle shaking. The gel was then destained using purified distilled water rinses until the water remained colorless.

### Gene expression measurement by quantitative real time PCR (qRT-PCR)

Total RNA was isolated from cells with the RNeasy minikit (Qiagen). RNA concentrations were quantified using the NanoDrop ND-1000 UV/Vis spectrophotometer and samples were normalized to 2 µg RNA. Reverse transcription was performed with 2 µg RNA, 210 ng random hexamers, 500 µM dNTPs, 84 units RNaseOUT (Thermo Fisher), and 210 units of Superscript II reverse transcriptase (Thermo Fisher) and incubated at 42°C for 55 min. qPCR reactions were run with 100 ng total input RNA, 1 µl (which contained 250 nM of the probe and 900 nM of the primers) of either FAM-labeled PARN (TaqMan Gene Expression Assay Hs00377733_m1 4331182, Thermo Fisher) and or FAM-labeled PLD2 (TaqMan Gene Expression Assay Hs01093219_m1 4351372) gene expression assay multiplexed with the FAM-labeled housekeeping genes Actin (TaqMan Gene Expression Assay Hs01060665_g1 4331182), GAPDH (TaqMan Gene Expression Assay Hs02758991_g1 4331182), and TATA-binding protein (TaqMan Gene Expression Assay Hs00427621_m1 4331182). qRT-PCR conditions for the Stratagene Mx3000P were: 95°C for 3 min and then 40 cycles of the next 3 steps: 30 s 95°C, 1 min 60°C, and then 1 min 72°C. The ‘cycle threshold’ Ct values were arbitrarily chosen from the linear part of the PCR amplification curve where an increase in fluorescence can be detected >10 s.e.m. above the background signal. ΔCt was calculated as: ΔCt=mean PLD Ct–mean housekeeping Ct; and gene fold-expression, as 2^-(ΔΔCt)^=2^-(experimental condition ΔCt – control ΔCt)^.

### Immunofluorescence microscopy

COS-7 cells were seeded onto sterilized coverslips placed in the bottom of the wells of 6-well plates and allowed 24 h for adherence. Cells were incubated for the times indicated in the figure legends in 30 nM fluorescent NBD-PA. Media was aspirated off of cells and cells gently rinsed once with 1× PBS. Cells were then fixed onto their cover slips using 4% paraformaldehyde for 10 min at room temperature. If only visualizing the NDB-PA, the nuclei were then stained with DAPI and the coverslips mounted to microscope slides as described further in the methods. If the cells were to be probed further with antibodies against proteins, the cells were then permeabilized with 0.5% Triton X-100 in PBS for 10 min at room temp and then incubated in 10% fetal calf serum (FCS)–0.1% Triton X-100 in PBS for 1 h at room temp. Endogenous expression of PARN was detected in samples using a 1:200 dilution of the corresponding IgG for 1 h at room temperature, followed by washing three times in 1× PBS, and further room temperature incubation with a 1:200 dilution of the appropriate TRITC-conjugated IgG secondary antibody for 1 h. For PARN, donkey anti-goat secondary antibody was used. After washing three times in 1× PBS, nuclei were stained using at a 1:2000 dilution of 4,6-diamidino-2-phenylindole (DAPI) in 1× PBS for 5 min at room temp. Coverslips were washed again three times in 1× PBS and once in distilled autoclaved water. Cover slips were then mounted onto clean glass microscope slides using VectaShield mounting medium, and cells were visualized using a Nikon 50 Eclipse epifluorescence microscope.

### Quantification of co-localization of PARN with PA

COS-7 cells were incubated for 30 min in 30 nM fluorescent PA (1-oleoyl-2-(6-[(7-nitro-2-1,3-benzoxadiazol-4-yl)amino]hexanoyl)-*sn*-glycero-3-phosphate, NBD-PA) and taken for immunofluorescence microscopy using TRITC-conjugated antibodies against PARN and DAPI. Photos were taken from separate fields under green (excitation=490 nm; emission=525 nm, FITC) and red (excitation=557 nm; emission=576 nm, TRITC) filters. Localization of the NBD-PA is in green fluorescence, localization of PARN is in red fluorescence and DAPI in deep blue fluorescence. Images were merged in Adobe Photoshop that rendered yellow spots associated with different sized intracellular vesicles. PA/PARN co-localization was represented by the presence of yellow (defined as 550 nm wavelength) in large (>0.5 mm vesicles), punctae (<0.5 mm vesicles) or diffuse distributions. This classification of vesicles is in accordance with a previous publication from our lab (Henkels et al., 2016). For quantification, relative fluorescence units (RFU) were measured from the image fields. The ratio for yellow (550 nm) was calculated with a method modified from (Comeau et al., 2006), as ratio (RFU_Yellow_-RFU_Red_)/total fluorescence in the field of observation and ratio (RFU_Yellow_-RFU_Green_)/total fluorescence in the field of observation. Co-localization occurs when the two ratio values are within 15% of each other and each of the ratios is at least 70% of maximum values.

### PLD and PARN activity assay

Cell lysates were processed for PLD activity in PC8 liposomes and [^3^H]n-butanol beginning with the addition of the following reagents (final concentrations): 3.5 mM PC8 phospholipid, 45 mM HEPES (pH 7.8) and 1.0µCi [3H]n-butanol in a liposome form, to accomplish the transphosphatidylation reaction of PLD, and were incubated for 20 min at 30°C with continuous shaking. Reactions were stopped with the addition of 0.3 ml ice-cold chloroform/methanol (1:2), and lipids were isolated and resolved by thin layer chromatography. The amount of [^3^H]-phospho-butanol ([^3^H]-PBut) that co-migrated with PBut standards (Rf=0.45–0.50) was measured by scintillation spectrometry.

For PARN activity, the *in vitro* assay was based on (Wilson and Treisman, 1988b; Shyu et al., 1991) with some modifications. Cell lysates overexpressing PARN or several other plasmids, as indicated in the legends to the figures, were subjected to deadenylation of a radiolabeled A_15_ RNA substrate. Positive controls were recombinant purified PARN and negative controls were just A_15_ substrate. Reactions products were subjected to autoradiography. Radiolabeled A_15_ RNA substrate was deadenylated by recombinant PARN or by COS-7 cell lysates in a reaction assay buffer contained protease and phosphatase inhibitors. Deadenylation is evidenced by a greater mobility of radiolabeled spots, the appearance of smears versus the negative control of A_15_ alone, and a lighter signal. This was quantified with a formula: PARN activity = 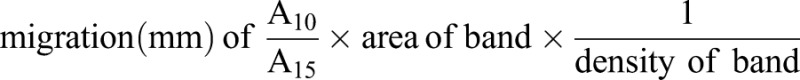.

### Statistical analysis

Data presented in the figures as bars are means+s.e.m. (standard deviation/*n*^1/2^, where *n* is the sample size). Experiments were performed in technical triplicates (for qPCR assays) or technical duplicates (for PARN and PLD activity assays) for *n*=5 independent experiments. The difference between means was assessed by the single factor analysis of variance (ANOVA) test, calculated using SigmaPlot version 10 (Systat Software Inc., San Jose, CA). Probability of *P*<0.05 indicates a significant difference. In the figures, the (*) symbols above bars denote statistically significant (*P*<0.05) ANOVA increases between samples and controls. The (#) symbols above bars denote statistically significant (*P*<0.05) ANOVA decreases between samples and controls.
